# Stabilisation of Bromenium Ions in Macrocyclic Halogen Bond Complexes

**DOI:** 10.1002/anie.202417427

**Published:** 2024-11-14

**Authors:** Andrew Docker, Heike Kuhn, Paul D. Beer

**Affiliations:** ^1^ Yusuf Hamied Department of Chemistry University of Cambridge Lensfield Road Cambridge CB2 1EW U.K.; ^2^ Chemistry Research Laboratory Oxford Department of Chemistry University of Oxford Mansfield Road Oxford OX1 3TA U.K.

**Keywords:** Halogen Bonding, Halogen(I) Complexes, Bromonium, Macrocyclic Effect, Halenium Ions

## Abstract

Halenium ions (X^+^) are highly reactive electron deficient species that are prevalent transient intermediates in halogenation reactions. The stabilisation of these species is especially challenging, with the most common approach to sequester reactivity through the formation of bis‐pyridine (Py) complexes; [(Py)_2_X]^+^. Herein, we present the first example of a macrocyclic stabilisation effect for halenium species. Exploiting a series of bis‐pyridine macrocycles, we demonstrate that preorganised macrocyclic ligands stabilise bromenium cations via endotopic complexation, impressively facilitating the isolation of a bench stable ‘Br^+^ NO_3_^−^’ species. Solid state X‐ray crystallographic structural comparison of macrocyclic Br(I) complexes with Ag(I) and Au(I) analogues provides insightful information concerning similarities and stark contrasts in halenium/metal cation coordination behaviors. Furthermore, the first chemical ligand exchange reactions of Br(I) complexes are reported between acyclic [(Py)_2_Br]^+^ species and a bis‐pyridine macrocyclic donor ligand which importantly highlights a macrocycle effect for halenium cation stabilisation in the solution phase.

## Graphical abstract

The first evidence of a macrocyclic effect for halenium (X^+^) ions is reported. Exploiting a series of bis‐pyridine macrocycles, we demonstrate the selective endotopic complexation of Br(I) species, remarkably facilitating the isolation of a stable ‘Br^+^ NO_3_^−^’ analogue and the first report of chemical ligand exchange reactions of Br(I) complexes. XRD studies of the macrocyclic Br(I) complexes and Ag(I) and Au(I) analogues provide rare insight concerning halenium vs. metal cation coordination behaviors.

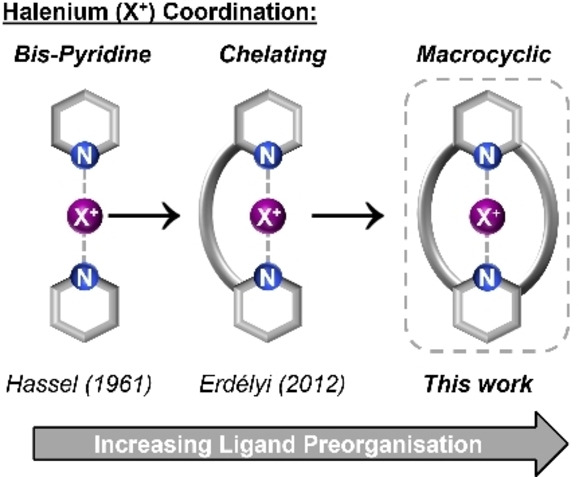

## Introduction

**Figure 1:**
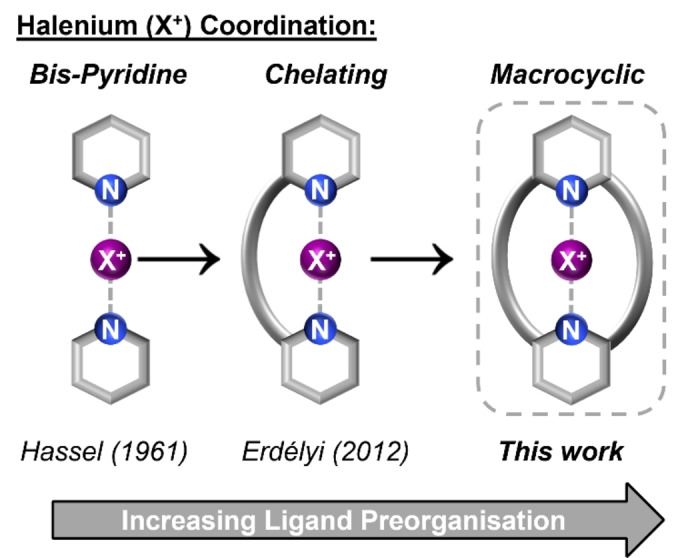
Cartoon representation of design conception.

Halogen Bonding (XB) describes the attractive interaction between an electron deficient region of a halogen atom and a Lewis base.^[1]^ Conceivably, the simplest XB donors would be positively charged halogen species or halenium ions (X^+^) and indeed X^+^ species are capable of interacting with two Lewis bases simultaneously. Halenium ions constitute some of the strongest XB donors to date,^[2]^ however, the high oxidising power and reactivity of X^+^ species often render them transient and elusive. The participation of X^+^ in XB interactions with Lewis bases can serve to stabilise their hypovalent nature, however this can also induce chemical transformation of the nucleophilic Lewis base. One successful strategy in ‘trapping’ X^+^ is through neutral nitrogenous bases, typically pyridine (Py), which generally form a three‐center, four‐electron (3c4e) bond via a linear [N⋅⋅⋅X⋅⋅⋅N]^+^ array. Arguably previously sporadic in report,[^3^, ^4^, ^5^, ^6^, ^7^] Erdélyi and Rissanen have pioneered the systematic study of halogen(I) complexes including those of the [(Py)_2_X]^+^ type^[8]^ and related bis(pyridin‐2‐ylethynyl)benzene chelating ligands,[^2^, ^9^] extensively characterising the effects of ligand electronics,^[10]^ influence of halogen identity^[11]^ and nature of the bonding interaction in these complexes (Figure 1).[^12^, ^13^, ^14^, ^15^, ^16^, ^17^, ^18^, ^19^, ^20^, ^21^, ^22^, ^23^, ^24^, ^25^, ^26^, ^27^, ^28^] As a consequence of periodic trends, halogen(I) complexes of this type exhibit serially decreasing stability for iodine, bromine and chlorine respectively and therefore the overwhelming majority of study has been directed towards iodine(I) complexes. From a coordination perspective, the high‐fidelity linear bonding arrangement of [N⋅⋅⋅X⋅⋅⋅N]^+^ halogen(I) complexes provide an exotic main group analogue of closed shell d^10^ transition metal cations such as Ag(I), Au(I) and Hg(II) which also exhibit linear coordination modes.^[29]^ As such, Erdélyi, Rissanen and others have recently reported elegant examples of self‐assembled supramolecular architectures such as helicates and capsules exploiting XB‐directed iodine(I) templating structural components.[^30^, ^31^, ^32^, ^33^, ^34^, ^35^, ^36^, ^37^] In the field of supramolecular host–guest recognition, ligand preorganisation serves as powerful means to enhance binding affinity for a given target guest, and indeed macrocyclic hosts have demonstrated prodigious efficacy in this task.^[38]^ Furthermore, the encapsulation afforded by architectures of this type can provide pronounced stabilisation for otherwise highly reactive chemistries.[^39^, ^40^, ^41^, ^42^, ^43^] Herein, we report the first example of a macrocyclic ligand for the stabilisation of halenium ions. Exploiting a series of bis‐pyridine containing macrocycles, including both symmetric and asymmetric ligand systems of varying sizes and pyridine Lewis basicity, through oxygen atom incorporation into the alkyl chain linkages, we demonstrate a ‘macrocyclic effect’ for halenium ions. This supramolecular host–guest stabilisation strategy provides solid state structural information for a library of bromine(I) macrocycle complexes, wherein structural comparison with Ag(I) and Au(I) analogues provides valuable insights into the coordination behavior of Br(I). This macrocyclic effect for Br(I) species stabilisation is also demonstrated in solution phase with the first report of chemical ligand exchange reactions for bromine(I) complexes.

## Results and Discussion

### Design and Synthesis of Bis‐Pyridine‐Based Macrocycles

**Scheme 1:**
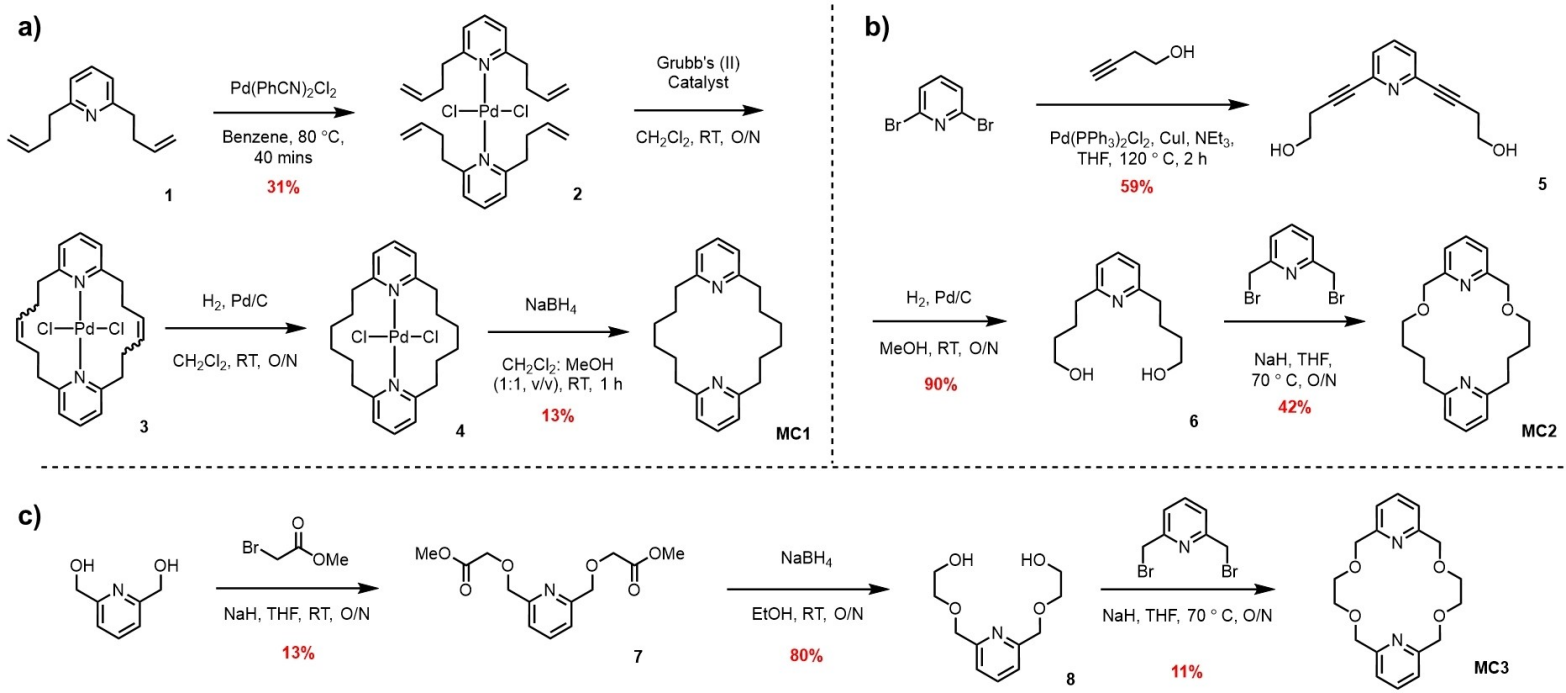
Synthetic routes towards the three target macrocycles a) **MC1** b) **MC2** c) **MC3**.

The general bis‐pyridine macrocycle design was conceived such that two aromatic pyridine units connected by alkyl linkers at their 2,6‐positions would generate a macrocyclic cavity and the opportunity for endotopic convergent N‐donor ligation of X^+^ species. Integration of oxygen donor atoms adjacent to the pyridyl methylene linkers would serve to modulate the Lewis base donor properties of the N‐donor atom and provide a synthetically accessible means by which to vary macrocyclic ligation properties. To this end, three bis‐pyridine macrocycles **MC1**, **MC2** and **MC3** were targeted (Scheme 1). The symmetric pyridinophane macrocycle, **MC1**, was prepared according to a modified literature procedure (Scheme 1a), in which 2,6‐diallylpyridine **1**, was subjected to complexation with bis(benzonitrile)palladium(II) chloride to afford the tetra‐allyl appended trans‐pyridine Pd(II) complex, **2**, in a yield of 31 % after purification by chromatography. A Grubbs’ II catalyst promoted double alkene metathesis macrocyclisation reaction of **2** in dichloromethane afforded **3** in quantitative yield. Hydrogenation of **3** using Pd/C in a H_2_ atmosphere gave **4**, the stability of the Pd(II) complex necessitated treatment with NaBH_4_ to achieve metal decomplexation which after chromatographic purification gave **MC1** in 13 % yield over three steps. The macrocyclic ligands **MC2** and **MC3** were prepared via Williamson ether synthesis macrocyclisation reactions. In the case of **MC2**, the requisite diol was prepared by a Sonogashira coupling reaction between 2,6‐dibromopyridine and 3‐butyn‐1‐ol, which gave **5** in a 59 % yield, following which an alkyne hydrogenation gave diol appended pyridine **6** in 90 % yield. For the synthesis of **MC3**, 2,6‐bis(hydroxymethyl)pyridine and methyl bromoacetate were treated with sodium hydride in anhydrous THF to afford bis‐ester **7** which after reduction with NaBH_4_ gave diol **8** in 80 % yield. Macrocylisation was achieved through dropwise addition of the appropriate diol, **6** or **8** and 2,6‐bis(bromomethyl)pyridine to a refluxing suspension of NaH in THF. Following column chromatography the macrocycles **MC2** and **MC3** were isolated in yields of 42 % and 11 % respectively. All three macrocycles were characterised by ^1^H and ^13^C NMR and high‐resolution electrospray ionization mass spectrometry (ESI‐MS).

### Formation of Ag(I) and Au(I) d^10^ Metal Complexes

**Scheme 2:**
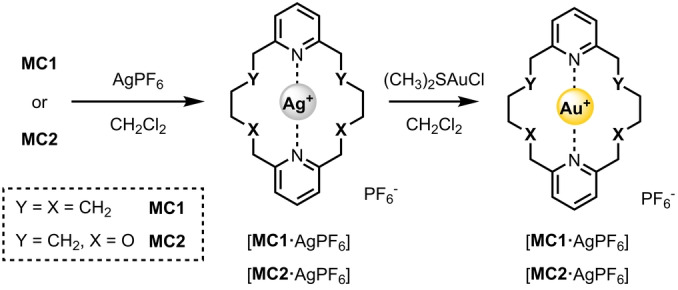
Synthesis of Ag(I) and Au(I) complexes of **MC1** and **MC2**.

**Figure 2:**
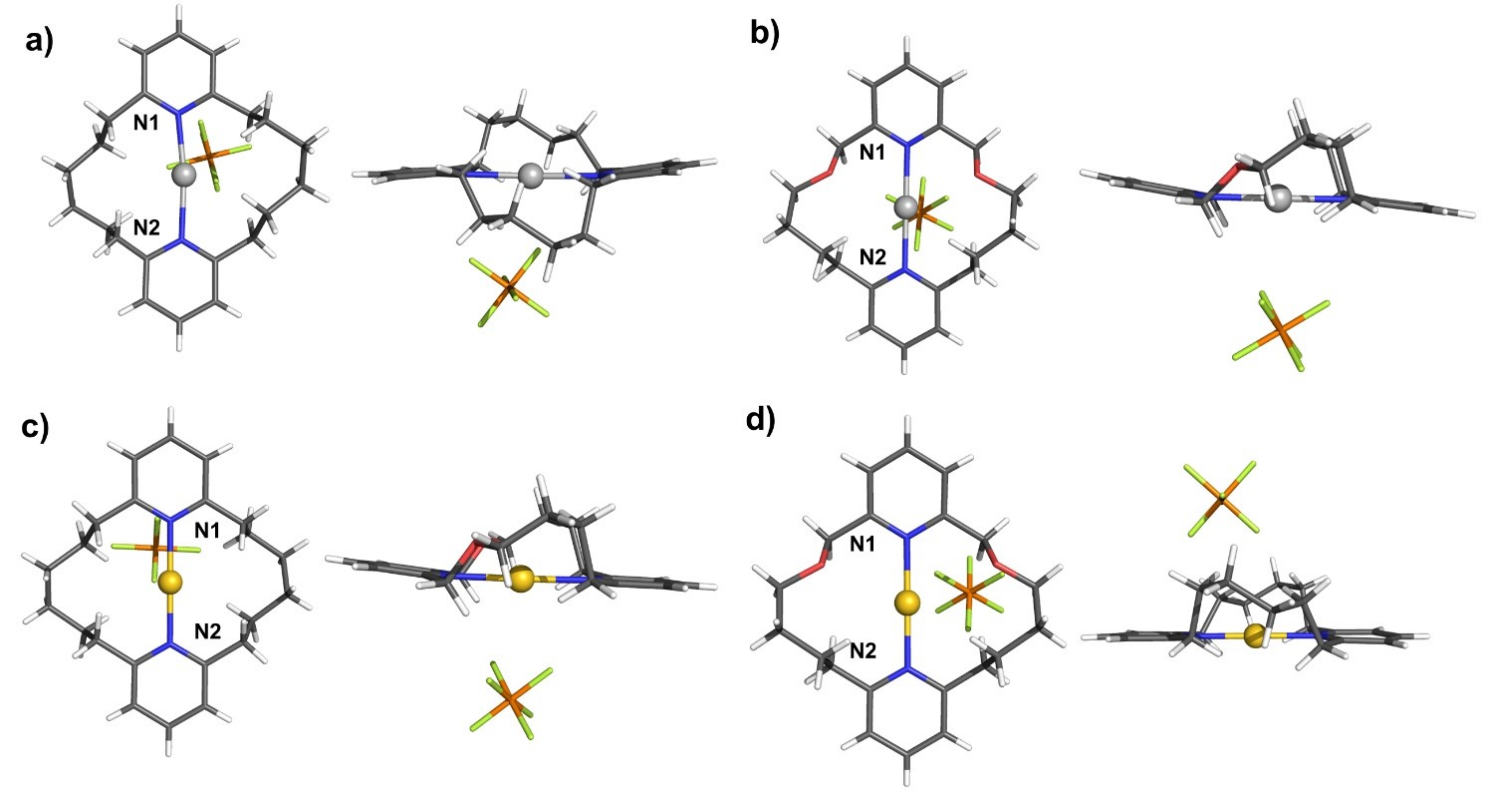
X‐ray single crystal structures with two different perspectives of a) [**MC1** ⋅ AgPF_6_] b) [**MC2** ⋅ AgPF_6_] c) [**MC1** ⋅ AuPF_6_] d) [**MC2** ⋅ AuPF_6_]. Grey=carbon, blue=nitrogen, white=hydrogen, green=fluorine, orange=phosphorus, red=oxygen, light grey=silver, yellow=gold.

With the target macrocycles in hand, attention was directed towards investigating their ligation properties. Both Ag(I) and Au(I) can be regarded as analogues of halenium cations, due to their electronic configuration and propensity to form two coordinate linear complexes. Furthermore, Ag(I) complexes are often used as precursors for the preparation of X^+^ species via their reaction with elemental halogens.[^9^, ^44^] Considering, the highly challenging nature of characterising reactive halogen(I) complexes by ^1^H NMR spectroscopy, we sought to identify macrocycles in the series which would facilitate unambiguous characterisation through single‐crystal X‐ray diffraction analysis. Indeed Erdélyi and co‐workers have identified several reports of purported halogen(I) complexes in which water‐induced decomposition has been established. To this end, Ag(I) complexation experiments were undertaken, in which **MC1**, **MC2** or **MC3** were dissolved in anhydrous CH_2_Cl_2_ together with an equimolar mixture of AgPF_6_ (Scheme 2). Whilst ^1^H NMR evidence for complexation was obtained for all macrocycles in the series, exhibiting spectra consistent with a linear coordination mode of Ag^+^, only **MC1** and **MC2** consistently gave high quality single crystals suitable for X‐ray diffraction (Figure 2a and 2b). As anticipated the solid‐state structures of [**MC1** ⋅ AgPF_6_] and [**MC2** ⋅ AgPF_6_] revealed the silver cation is bound endotopically by forming two N⋅⋅⋅Ag coordinate bonds. Selecting **MC1** and **MC2** as suitable candidates for further study, their Au(I) complexes were also prepared via a transmetalation procedure (Scheme 2). The corresponding Ag(I) complexed macrocycles, were treated with a Au(I) source; (CH_3_)_2_SAuCl, which immediately induced AgCl precipitation and Au(I) complexation. Solid state X‐ray diffraction structural determination of the resultant [**MC1** ⋅ AuPF_6_] and [**MC2** ⋅ AuPF_6_] complexes similarly confirmed the endotopic coordination of the gold cation via two linear N⋅⋅⋅Au coordinate bonds (Figure 2c and 2d). A summary of the selected distances and angles for the four complexes is detailed in Table 1. Inspection of Table 1 reveals [**MC1** ⋅ AgPF_6_] exhibits very moderate asymmetry with N⋅⋅⋅Ag^+^ distances of 2.113 Å and 2.235 Å, whilst [**MC2** ⋅ AgPF_6_] possesses much more similar N⋅⋅⋅Au^+^ distances of 2.188 Å and 2.183 Å. Interestingly, the incorporation of oxygen into the macrocycle scaffold of **MC2** does not seem to influence symmetry of silver(I) ligation, whilst the all‐carbon analogue, **MC1**, does exhibit unequal N⋅⋅⋅Ag^+^ distances. Similar comparisons for the gold(I) complexes reveal highly symmetric N⋅⋅⋅Au^+^⋅⋅⋅N distances for both macrocycles, albeit moderately contracted for **MC1** relative to **MC2**. It should also be noted that stable bis‐pyridine complexes of Au(I) are themselves very rare and readily prone to disproportionation, highlighting the marked stability of these complexes which is presumably due to a macrocyclic effect.

**Table 1:** Selected distances and angles calculated from the solid‐state structures of [**MC1** ⋅ AgPF_6_], [**MC2** ⋅ AgPF_6_], [**MC1** ⋅ AuPF_6_], and [**MC2** ⋅ AuPF_6_] using PLATON. Values in angstroms or degrees. Standard uncertainties (±) are in parentheses. M^+^=Ag^+^ or Au^+^.

	**Distance (Å)**		**Angle (°)**
	M^+^⋅⋅⋅N1	M^+^⋅⋅⋅N2	N1⋅⋅⋅M^+^⋅⋅⋅N2
[**MC1** ⋅ AgPF_6_]	2.113(11)	2.235(6)	172.1(4)
[**MC2** ⋅ AgPF_6_]	2.188(3)	2.183(3)	179.56(9)
[**MC1** ⋅ AuPF_6_]	2.055(2)	2.060(2)	179.90(10)
[**MC2** ⋅ AuPF_6_]	2.079(4)	2.074(4)	178.94(14)

### Formation of Bromine(I) Complexes

**Figure 3:**
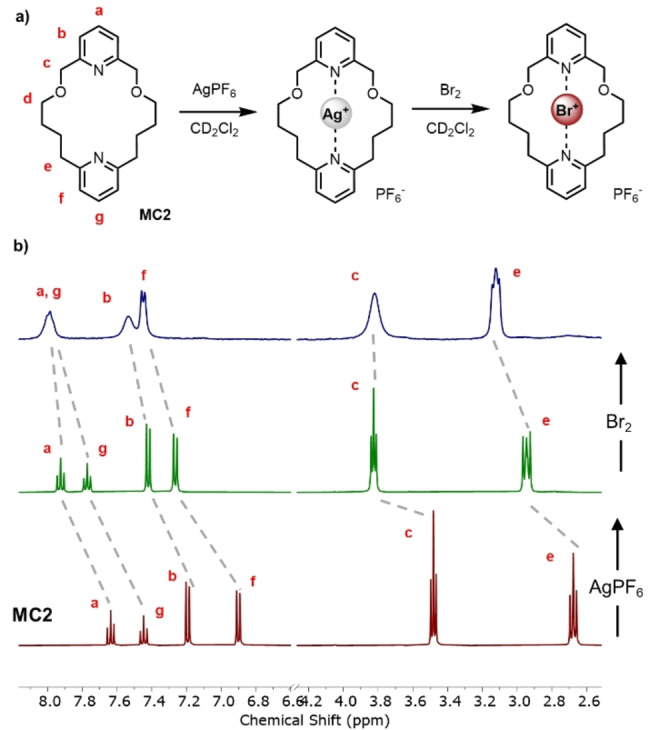
a) Bromine(I) complex formation scheme b) ^1^H NMR spectra of **MC2** (bottom), [**MC2** ⋅ AgPF_6_] (middle), and [**MC2** ⋅ BrPF_6_] (top). (CD_2_Cl_2_, 400 MHz, 298 K).

**Figure 4:**
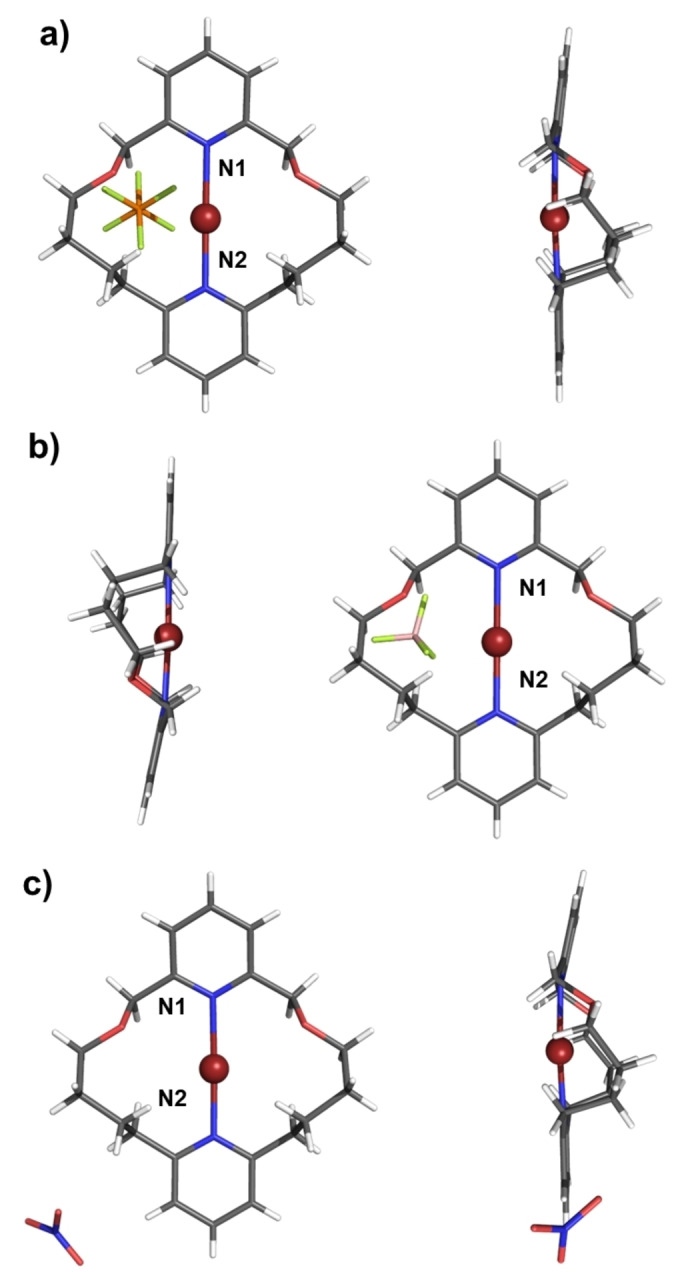
X‐ray single crystal structures of a) [**MC2** ⋅ BrPF_6_], b) [**MC2** ⋅ BrBF_4_] and c) [**MC2** ⋅ BrNO_3_] with two different perspectives. Grey=carbon, blue=nitrogen, white=hydrogen, green=fluorine, pink=boron orange=phosphorus, red=oxygen, brown=bromine.

With the ability of **MC1** and **MC2** to linearly coordinate Ag(I) and Au(I) d^10^ transition metal cations established, the macrocycles’ ability to complex halenium ions was subsequently investigated. The generation of halogen(I) complexes is typically achieved by treatment of a Ag(I) precursor complex with a source of elemental halogen, in which a disproportionation reaction occurs precipitating the corresponding silver halide. To this end, a ^1^H NMR titration experiment was undertaken, in which a CD_2_Cl_2_ solution of [**MC1** ⋅ AgPF_6_] or [**MC2** ⋅ AgPF_6_] was treated with I_2_. To our surprise, after several days at room temperature and despite heating, the purple colour of elemental iodine persisted suggesting iodine(I) formation had not occurred, this was further confirmed by negligible perturbation of the ^1^H NMR spectrum. Notwithstanding the apparent kinetic inertness of the Ag(I) complexes being responsible for the lack of reaction with I_2_, ambitiously the more reactive halogen Br_2_ was selected as the partner for the disproportionation reaction. Pleasingly, the addition of a Br_2_ solution in CD_2_Cl_2_ to [**MC1** ⋅ AgPF_6_] or [**MC2** ⋅ AgPF_6_] resulted in immediate precipitation of AgBr. The ^1^H NMR spectrum of the remaining solution revealed the formation of new species notably distinct from the macrocycle or the corresponding silver(I) complex, a representative example with **MC2** is shown in Figure 3. To our delight, approximately after three hours of standing at ambient temperature, single crystals suitable for X‐ray diffraction were obtained from a sample of [**MC2** ⋅ AgPF_6_] post Br_2_ addition, which indeed revealed the anticipated bromine(I) species, wherein ‘Br^+^’ is residing in the macrocycle interior stabilised through an endotopic N⋅⋅⋅Br^+^⋅⋅⋅N bonding arrangement (Figure 4a). Motivated by this, we sought to generate a library of Br(I), Ag(I) and Au(I) complexes with **MC2** varying the counterion nature. In a manner analogous to the aforementioned procedures the following complexes were prepared and structurally characterised; [**MC2** ⋅ BrBF_4_], [**MC2** ⋅ BrNO_3_] (Figure 4b and 4c), [**MC2** ⋅ AgBF_4_], [**MC2** ⋅ AgNO_3_], [**MC2** ⋅ AuBF_4_] and [**MC2** ⋅ AuNO_3_] selected structural details are summarized in Table 2.

**Table 2:** Selected distances calculated from the solid‐state structures of [**MC2** ⋅ BrPF_6_], [**MC2** ⋅ BrBF_4_], [**MC2** ⋅ BrNO_3_], [**MC2** ⋅ AgPF_6_], [**MC2** ⋅ AgBF_4_], [**MC2** ⋅ AgNO_3_], [**MC2** ⋅ AuPF_6_], [**MC2** ⋅ AuBF_4_] and [**MC2** ⋅ AuNO_3_] using PLATON. Values in angstroms or degrees. Standard uncertainties (±) are in parentheses.

	Distance (Å)					
X^+^ =	Br(I)		Ag(I)		Au(I)	
Counter Anion	Br^+^⋅⋅⋅N1	Br^+^⋅⋅⋅N2	Ag^+^⋅⋅⋅N1	Ag^+^⋅⋅⋅N2	Au^+^⋅⋅⋅N1	Au^+^⋅⋅⋅N2
PF_6_^−^	2.234(3)	2.099(3)	2.186(3)	2.183(3)	2.069(4)	2.076(4)
BF_4_^−^	2.243 (5)	2.096(5)	2.195(2)	2.193(2)	2.066(3)	2.068(3)
NO_3_^−^	2.227(2)	2.090(2)	2.238(1) 2.260(1) 2.243(1)	2.224(1) 2.234(1) 2.233(1)	2.089(4)	2.078(4)

In contrast to the highly symmetric Au(I) and Ag(I) complexes, Br(I) demonstrated pronounced asymmetry in the N⋅⋅⋅Br^+^⋅⋅⋅N bond lengths. For all three Br(I) complexes, the N⋅⋅⋅Br^+^ distance is consistently longer for N1 suggesting that the presence of the Lewis basic oxygens serves to ‘repel’ Br(I) centre. Whilst the exact origin of this effect, whether it is repulsion from the methylene oxygens or the reduced basicity of the pyridine nitrogen by the electron withdrawing methylene oxygens, is unclear, the N⋅⋅⋅Br^+^⋅⋅⋅N bond asymmetry of 6–7 % is evident and it is believed not to be solely due to crystal packing. The strong directionality of the N⋅⋅⋅Br^+^⋅⋅⋅N interaction and preference for linear bond formation was observed in all three bromine(I) complexes and is also shared with the Ag(I) and Au(I) complexes. The notable difference in the angle of the three symmetrically unrelated molecules in the asymmetric unit of [**MC2** ⋅ AgNO_3_] arises from the coordination of the NO_3_^−^ counter anion to the Ag(I) centre (Figure S14). This difference in the coordination of the nitrate anion to Ag(I) reflects one of the most fundamental differences between halenium and transition metal cation behaviours. Unlike its transition metal counterpart, the Br(I) centre is inert to coordination by additional Lewis bases in the form of the counter anion, as discussed by Erdélyi, where the series of Br(I) complexes remain structurally comparable despite the change in the coordinating ability of the counter anion.[^13^, ^45^] It should be mentioned that during isolation of ‘Br^+^ NO_3_^−^’ in the form of [**MC2** ⋅ BrNO_3_], the sample exhibited no signs of decomposition by ^1^H NMR over the course of 3 days at room temperature is starkly contrasted by the ready decomposition of bromine nitrate (BrNO_3_) above temperatures of 0 °C. Indeed, to the best of our knowledge not only does the above series constitute the largest collection of homologous structurally characterised bromine(I) complexes, barring those of [(Py)_2_Br]^+^ type, but also the first example of a stable bromine(I) complex with an anion as coordinating as nitrate. The three bromine(I) complexes reported here also displayed remarkable stability during manipulation, whilst previous reports typically describe decomposition when handling crystals of Br(I) complexes or during XRD experiments, this was not observed for the [**MC2** ⋅ Br]^+^ complexes.^[46]^

### Preliminary Investigation of a Halonium Cation Macrocyclic Effect

**Figure 5:**
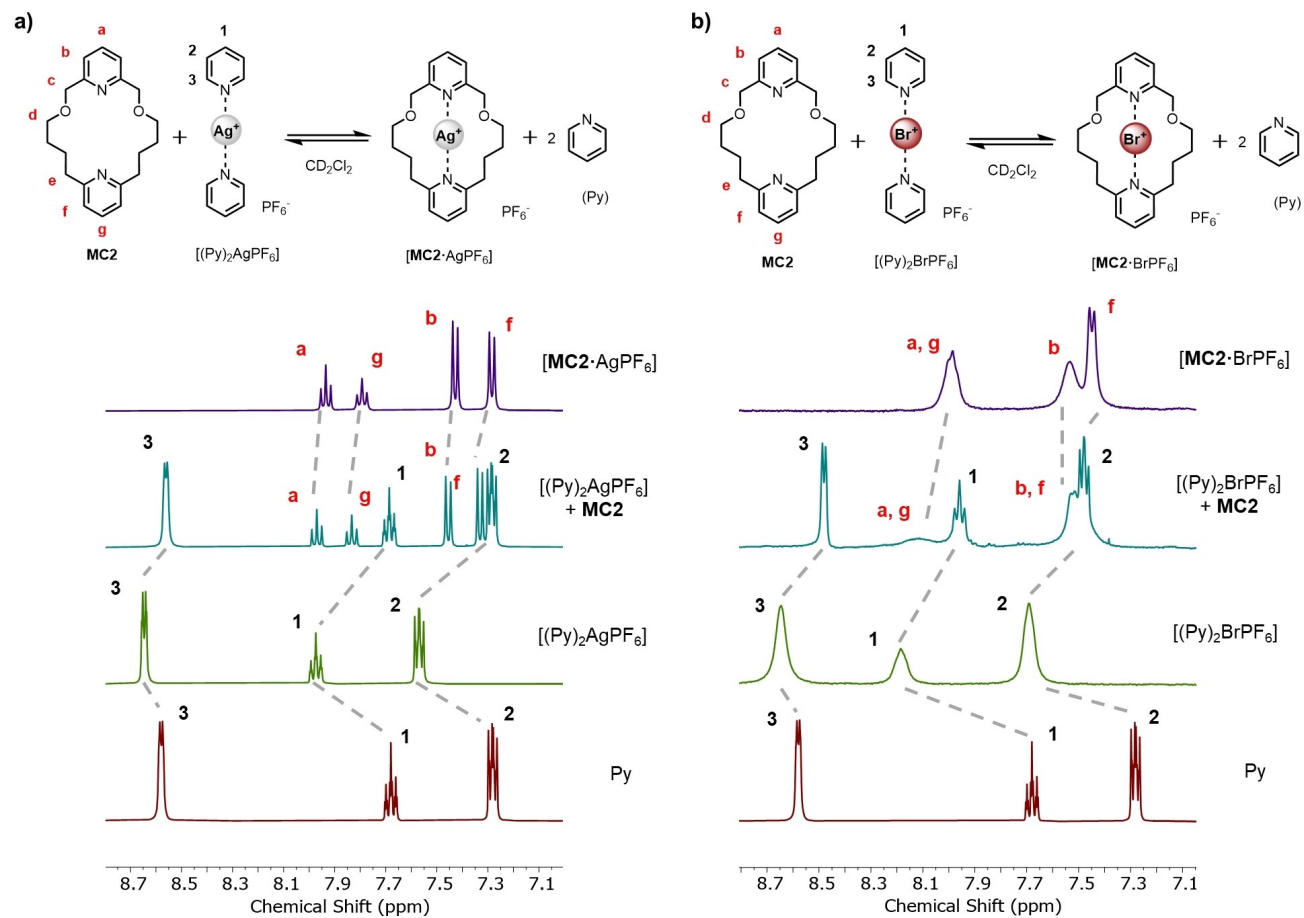
Truncated and stacked ^1^H NMR spectra of the exchange reaction reactions performed with **MC2** and a) [(Py)_2_AgPF_6_] and b) [(Py)_2_BrPF_6_] (CD_2_Cl_2_, 400 MHz, 298 K).

Having determined the solid‐state single crystal XRD structures of the **MC2** bromine(I) complexes and experimental evidence that the macrocyclic ligand **MC2** is responsible for the remarkable observed halenium cation stability, attention turned to investigate solution phase evidence of a macrocyclic effect for Br(I) complexation. It was postulated the macrocyclic nature of **MC2** would translate into enhanced thermodynamic stability of the bromine(I) complex relative to a simple acyclic [(Py)_2_Br]^+^ type complex. Firstly, it was necessary to confirm **MC2** would exhibit the predicted macrocyclic effect with a more handleable and less sensitive cation, namely Ag^+^. Therefore an exchange reaction was conducted, in which a preformed bis‐pyridine Ag(I) complex, [(Py)_2_Ag]^+^ PF_6_^−^, was mixed with an equimolar amount of the free macrocycle **MC2** (Figure 5a) in CD_2_Cl_2_ solution. Monitored by ^1^H NMR, as anticipated the post‐mixture spectrum indicated Ag^+^ exchange, wherein the silver cation is preferentially complexed by **MC2** and free pyridine is liberated. With this NMR evidence in hand for the enhanced stability of the [**MC2** ⋅ Ag]^+^ relative to an acyclic [(Py)_2_Ag]^+^ analogue, an analogous experiment was performed with an in situ generated [(Py)_2_Br]^+^ PF_6_^−^ complex (Figure 5b). Inspection of the ^1^H NMR spectrum of [(Py)_2_Br]^+^ reveals dramatically broadened signals relative to free pyridine and indeed this signal broadening is also observed with the independently prepared [**MC2** ⋅ BrPF_6_] complex, which is presumably due to scalar relaxation by the quadrupolar nuclei of bromine isotopes. Upon mixing the free macrocycle **MC2** and the generated [(Py)_2_Br]^+^ complex, well resolved signals corresponding to free pyridine and the characteristically broadened signals of the [**MC2** ⋅ Br]^+^ complex were observed. Indeed, whilst ligand exchange reactions have been recently documented for iodine(I) complexes, to the best of our knowledge this is the first example of chemical exchange for bromine(I) complexes. Whilst the spectral resolution and experimental challenge prevent accurate determination of the exchange ratios, an obvious Br(I) ligand exchange proceeded and considering the dramatic resolution observed in the proton signals of the liberated pyridine it seems to follow this indicates a preference for the macrocyclic host.

## Conclusions

In conclusion, we report the first macrocyclic ligands for Br(I) complexation and stabilisation. Targeting a series of bis‐pyridine integrated macrocyclic structural frameworks, we demonstrate through solid state X‐ray crystallographic structural analysis and solution phase ^1^H NMR investigations that Br(I) is endotopically coordinated through a stabilising N⋅⋅⋅Br^+^⋅⋅⋅N bonding array. Insight into the bonding characteristics of these species is obtained through extensive structural characterisation and comparison with a library of Br(I) complexes and their Ag(I) and Au(I) analogues. Indeed, whilst parallels in behaviour of Br(I), Ag(I) and Au(I) are often discussed, this study indicates they can possess stark differences. For example, whilst ligand asymmetry does not affect Ag(I) or Au(I) bonding symmetries it consistently influences the Br(I) complexes and whereas Ag(I) remains capable of Lewis base coordination, the Au(I) and Br(I) are consistently inert, highlighting fundamental differences in nature and character between these three‐centre four‐electron bonding systems. Furthermore, the stabilisation conferred by the macrocyclic nature of the ligand facilitates unprecedented isolation of the normally highly reactive and unstable ‘Br^+^ NO_3_^−^’ species. Significantly, we also demonstrate evidence for a solution phase macrocyclic effect via Br(I) complex chemical ligand exchange. Crucially, these results serve to highlight the established coordination chemistry concept of the macrocyclic effect is applicable to augmenting the stability of reactive halogen(I) species.

## Conflict of Interests

The authors declare no conflict of interest.

1

## Supporting information

As a service to our authors and readers, this journal provides supporting information supplied by the authors. Such materials are peer reviewed and may be re‐organized for online delivery, but are not copy‐edited or typeset. Technical support issues arising from supporting information (other than missing files) should be addressed to the authors.

Supporting Information

## Data Availability

The data that support the findings of this study are available in the supplementary material of this article.
