# Supplementary material for: Stabilisation of Bromenium Ions in Macrocyclic Halogen Bond Complexes
Source: Angew Chem Int Ed Engl. 2024 Nov 14;64(5):e202417427. doi: 10.1002/anie.202417427 (PMC11773099; doi:10.1002/anie.202417427)
Supplement: Supplementary file 1 — Supporting Information [file ANIE-64-e202417427-s001.pdf]

Angewandte Chemie
Eine Zeitschrift der Gesellschaft Deutscher Chemiker

Supporting Information

Stabilisation of Bromenium Ions in Macrocyclic Halogen Bond Complexes

A. Docker, H. Kuhn, P. D. Beer*

Supporting information for

Stabilisation of Bromenium Ions in Macrocyclic Halogen Bond Complexes

Andrew Docker,[a]* Heike Kuhn[b] and Paul D. Beer[b].

[a] Yusuf Hamied Department of Chemistry, University of Cambridge, Lensfield Road, Cambridge, CB2 1EW U.K.

[b] Chemistry Research Laboratory, Department of Chemistry, University of Oxford, Mansfield Road, Oxford OX1 3TA, U. K.

Contents

1. Materials and Methods 2

2. Synthetic Procedures and Characterisation 2

General Synthetic Procedures 2

Characterisation of Br(I) Ag(I) and Au(I) complexes 10

3. Crystal Structure Determination 12

1. Materials and Methods

All solvents and reagents were purchased from commercial suppliers and used as received unless otherwise stated. Dry solvents were obtained by purging with nitrogen and then passing through an MBraun MPSP-800 column. H_2O was de-ionized and micro filtered using a Milli-Q ® Millipore machine. Routine NMR spectra were recorded on either a Varian Mercury 300, a Bruker AVIII 400 or a Bruker AVIII 500 spectrometer with ^{1}H NMR titrations recorded on a Bruker AVIII 500 spectrometer.

2. Synthetic Procedures and Characterisation

General Synthetic Procedures

General Procedure 1: 2,6-dibromopyridine (1.00 g, 4.22 mmol, 1 eq.), Pd(PPh_3)Cl_2 (296 mg, 0.422 mmol, 0.1 eq.) and CuI (80 mg, 0.422 mmol, 0.1 eq.) were sealed into a microwave vial to which THF (8 ml) and NEt_3 (8 ml) were added and the mixture thoroughly sonicated. The appropriate alkyne (21.1 mmol, 5 eq.) was added and the reaction mixture was heated under microwave irradiation at 80°C for 1 hour. The crude mixture was then diluted with CH_2Cl_2 (ca. 100 ml) and filtered through celite. The filtrate was concentrated to a residue in vacuo and subsequently redissolved in CH_2Cl_2 (ca. 300 ml). The organic phase was washed with EDTA/NH_4OH(aq) solution (20 ml), then water (10 ml), dried over $MgSO_4$ and the solvent removed in vacuo. The crude residue was purified by silica gel column chromatography (EtOAc/MeOH mixtures) to afford the product.

General Procedure 2: The appropriate alkyne-diol (4.00 mmol) was dissolved in MeOH (50 ml), to which was carefully added 10% Pd/C (10% w/w), the reaction was then subjected to an atmosphere of hydrogen and the reaction mixtures stirred overnight at room temperature. The mixture was then filtered and concentrated in vacuo to afford the appropriate diol.

General Procedure 3: NaH (60 % /w dispersion) was suspended in anhydrous THF (100 ml) and stirred at 70°C for 30 minutes. Separate solutions of 2,6-bis(bromomethyl)pyridine (1.32 g, 4.98 mmol, 1 eq.) and the appropriate diol (4.98 mmol, 1 eq.), each in THF (50 ml), were added dropwise to the refluxing solution over the course of 3 hours. Once the addition was complete, the mixture was left to stir overnight at 70°C. After which, the mixture was left to cool to room temperature and was carefully quenched by the addition of MeOH (ca. 50 ml). The resultant mixture was concentrated to dryness in vacuo and the crude reaction mixture was partitioned between CH_2Cl_2 (250 ml) and H_2O (250 ml), the organic phase was collected and washed with H2O (250 ml) dried over $MgSO_4$ and the solvent removed in vacuo. The crude reaction mixture was subjected to silica gel chromatography (3:2:0.3, CH_2Cl_2/EtOAc/MeOH) to afford the product.

General Procedure 4: The respective macrocycle (0.046 mmol, 1 eq.) and the appropriate silver salt (0.046 mmol, 1 eq.) were dissolved in anhydrous CH_2Cl_2 (ca. 2 ml) and left to stir for 1 hour. After this the solution was filtered to yield a solution of the silver complex.

General Procedure 5: The respective macrocycle (0.046 mmol, 1 eq.) and the appropriate silver salt (0.046 mmol, 1 eq.) were dissolved in anhydrous CH_2Cl_2 (ca. 2 ml) and left to stir

for 1 hour. To this stirred solution was added $(Me_2S)AuCl$ (13.5 mg , 0.046 mmol, 1 eq.) after which time AgCl immediately precipitated and left to stir for 30 minutes. After this the solution was filtered to yield a solution of the gold(I) complex.

General Procedure 6: The respective macrocycle (15 mg, 0.046 mmol, 1 eq.) and the appropriate silver salt (0.046 mmol, 1 eq.) were dissolved in anhydrous CH_2Cl_2 (ca. 2 ml) and left to stir for 1 hour. An anhydrous 1 M Br_2 in CH_2Cl_2 solution (0.046 mmol) was added dropwise the stirred solution, after which time AgBr immediately precipitated and left to stir for 30 minutes. After this the solution was filtered to yield a solution of the bromonium complex.

Compounds **1**, **2**, **3** and **4** were synthesized according to literature procedures and spectroscopic data were consistent with those reported.[1]

Compounds **6**, **7**, **8** and **MC3** were synthesized according to literature procedures and spectroscopic data were consistent with those reported.[2]

Compound **MC3** was synthesized according to general procedure 3 and spectroscopic data were consistent with those reported.[3]

Compound **5**

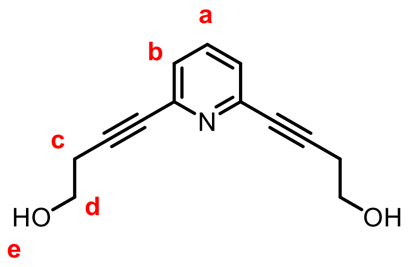

Prepared according to general procedure 1 from commercially available 3-butyn-1-ol and 2,6 dibromopyridine and purified by silica gel flash column chromatography (MeOH:EtOAc) to afford the product as a yellow solid in 59% yield. **^{1}H NMR** (400 MHz, Acetone-d_6) δ 7.71 (t, J = 7.8 Hz, 1H, H_a), 7.35 (d, J = 7.8 Hz, 2H, H_b), 4.06 (t, J = 6.0 Hz, 2H, H_e), 3.75 (td, J = 6.8, 6.0 Hz, 4H, H_d), 2.63 (t, J = 6.8 Hz, 4H, H_c). **^{13}C NMR-{^{1}H}** (101 MHz, Acetone-d_6) δ 144.64, 137.57, 126.54, 88.97, 81.70, 61.10, 24.18. **HRESI-MS** (pos.) m/z 216.1017 [MH^+] calc. for [$C_{13}H_{13}NO_2{\cdot}H^+$] 216.1019.

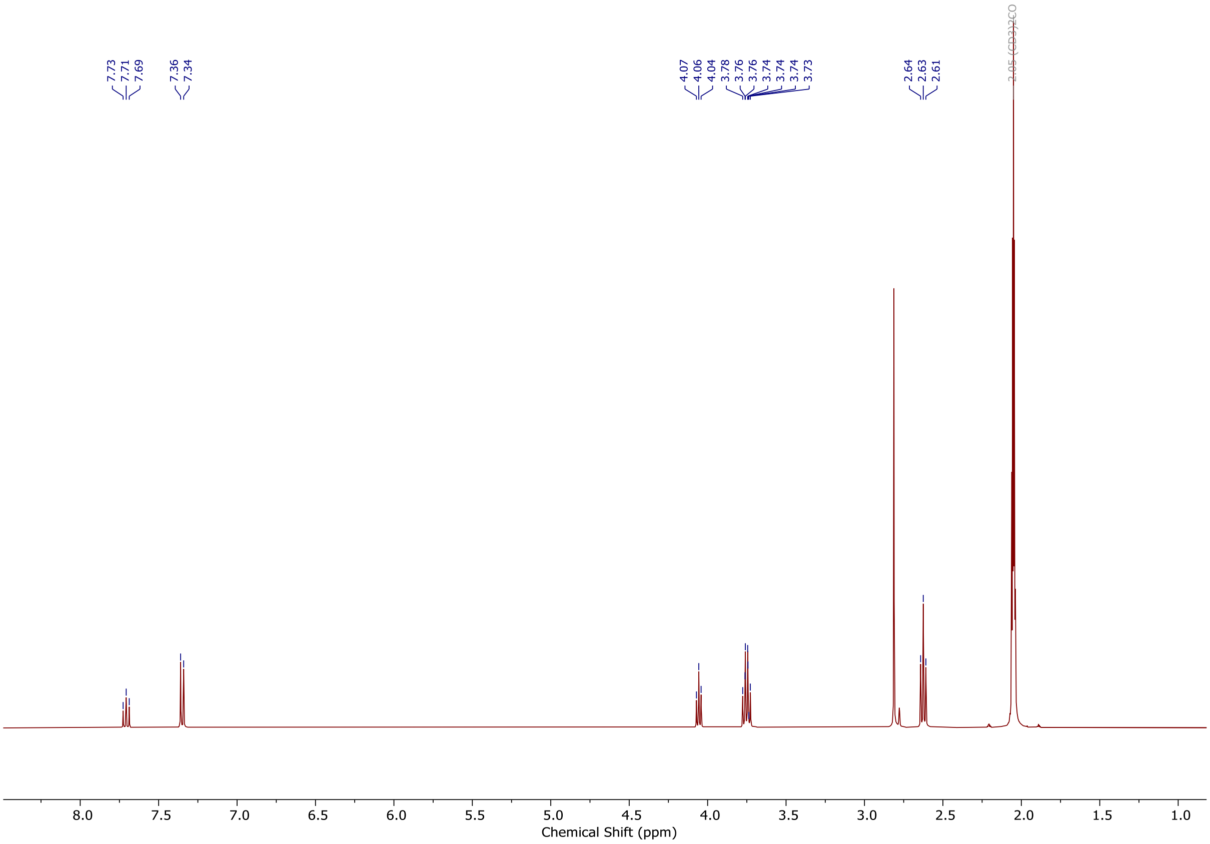

Figure S1. ^{1}H NMR spectrum of X (acetone-d_6, 400 MHz, 298 K).

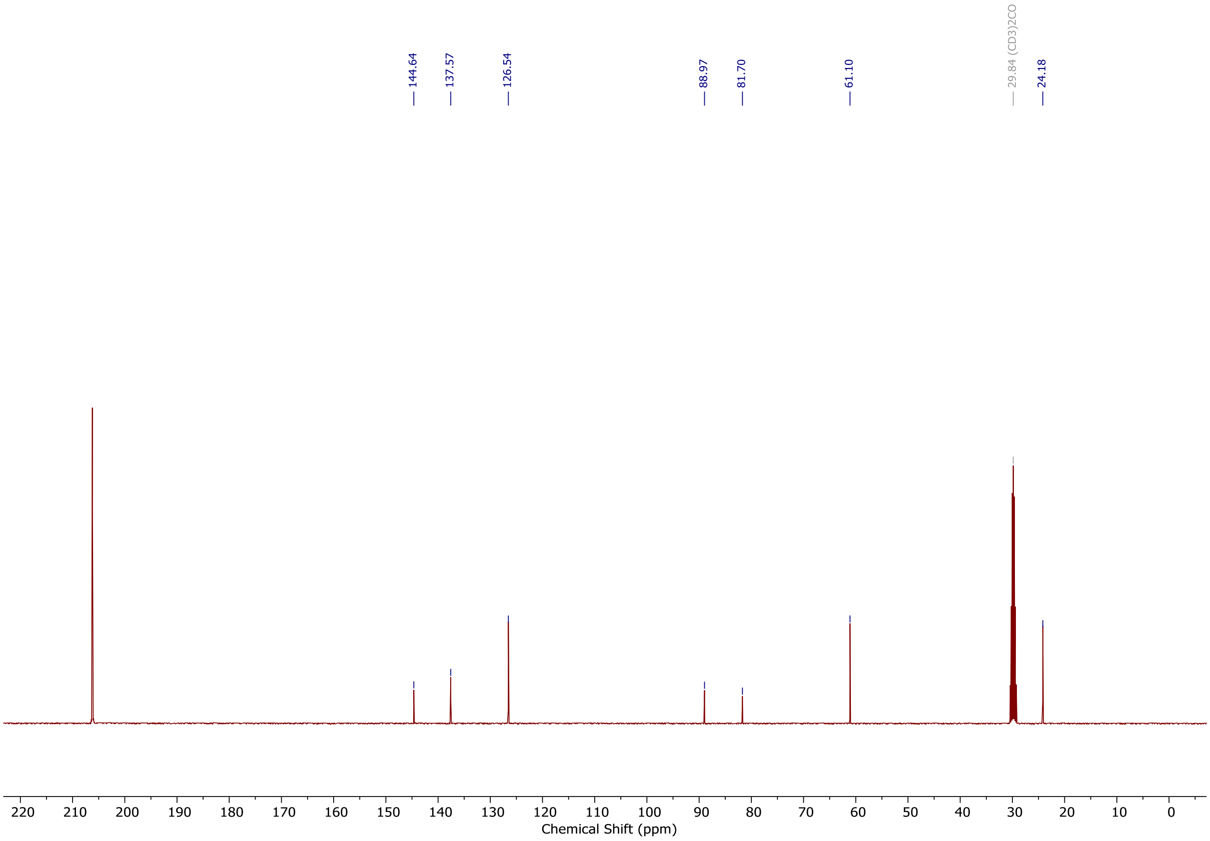

Figure S2. ^{13}C NMR spectrum of X (acetone-d_6, 101 MHz, 298 K).

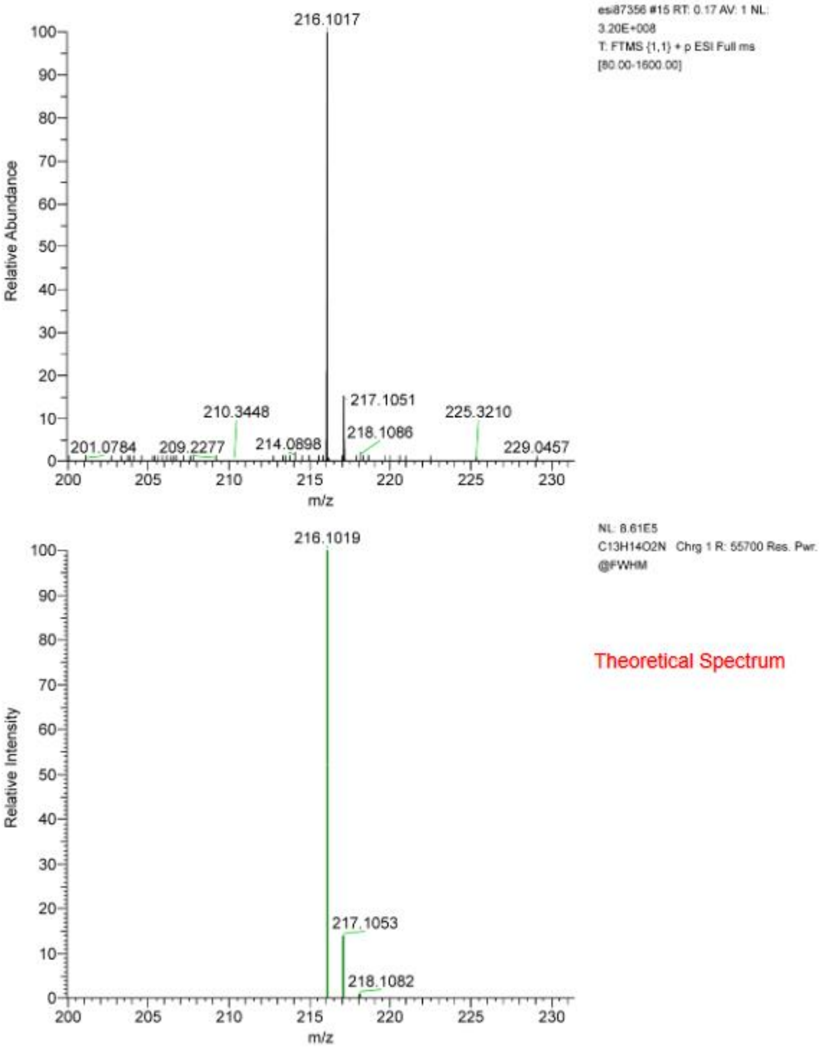

Figure S3. HRESI spectrum of X.

Compound **6**

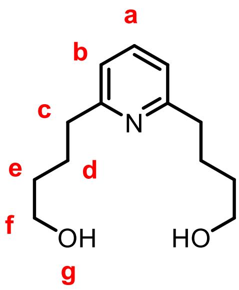

Prepared according to general procedure 2 from 5 to afford the product as a colourless oil in 78% yield. **^{1}H NMR** (400 MHz, Acetone-d_6) δ 7.55 (t, J = 7.7 Hz, 1H, Ha), 7.02 (d, J = 7.7 Hz, 2H, Hb), 3.63 (s, 2Hg), 3.57 (t, J = 6.5 Hz, 4H, Hf), 2.91 – 2.59 (m, 4H, Hc), 1.89 – 1.68 (m, 4H, He), 1.66 – 1.44 (m, 4H, Hd). **^{13}C NMR-{^{1}H}** (101 MHz, Acetone-d_6) δ 162.30, 137.30, 120.55, 62.25, 38.50, 33.32, 26.92. **HRESI-MS** (pos.) m/z 224.1647 [MH$^+$] calc. for [$C_{13}H_{21}NO_2{\cdot}H^+$] 224.1645.

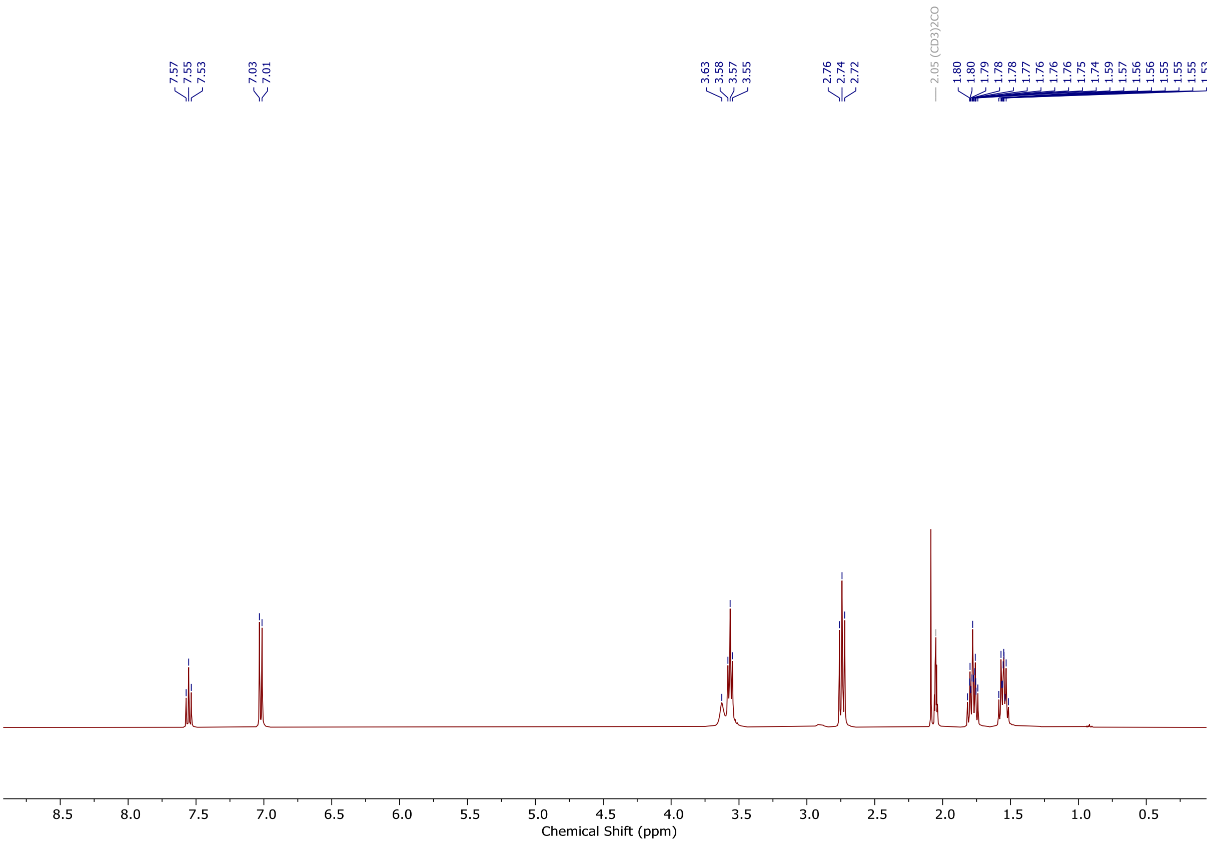

Figure S4. ^{1}H NMR spectrum of X (acetone-d_6, 400 MHz, 298 K).

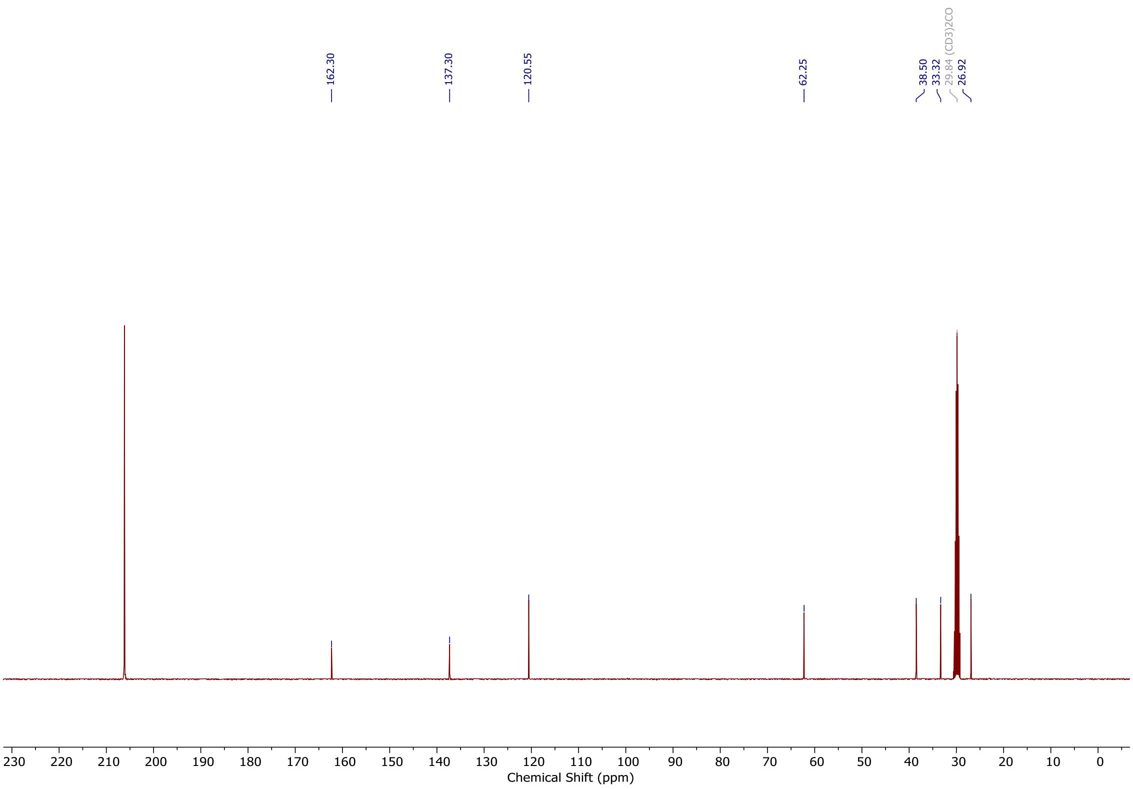

Figure S5. ^{13}C NMR spectrum of X (acetone-d_6, 101 MHz, 298 K).

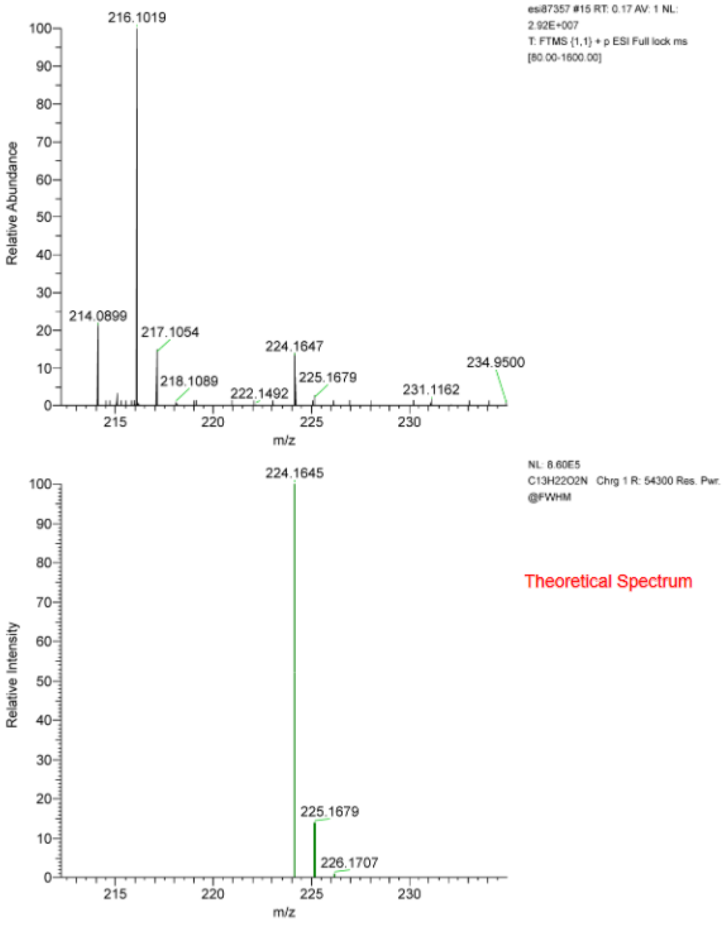

Figure S6. HRESI spectrum of X.

Compound **MC2**

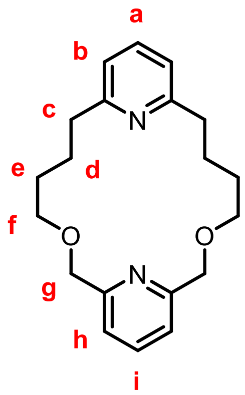

Prepared according to general procedure 3 from 6 and 2,6-bis(bromomethyl)pyridine and subjected to silica gel flash column chromatography (3:2:0.3 CH_2Cl_2/EtOAc/MeOH) to afford the product as white solid in 42% yield. **^{1}H NMR** (400 MHz, Acetone-d_6) δ 7.71 (t, J = 7.6 Hz, 1H, Hi), 7.50 (t, J = 7.6 Hz, 1H, Ha), 7.26 (d, J = 7.6 Hz, 2H, Hh), 6.96 (d, J = 7.6 Hz, 2H, Hb), 4.45 (s, 4Hg), 3.51 (t, J = 6.1 Hz, 4Hc), 2.67 (t, J = 7.4 Hz, 4Hf), 1.79 (p, J = 7.5 Hz, 4Hd), 1.66 – 1.41 (m, 4He). **^{13}C NMR-{^{1}H}** (101 MHz, Acetone-d_6) δ 162.09, 159.06, 137.48, 137.07, 122.09, 120.65, 73.54, 70.15, 38.12, 26.69. **HRESI-MS** (pos.) m/z 327.2062 [MH^+] calc. for [$C_{20}H_{26}N_2O_2 \cdot H^+$] 327.2067.

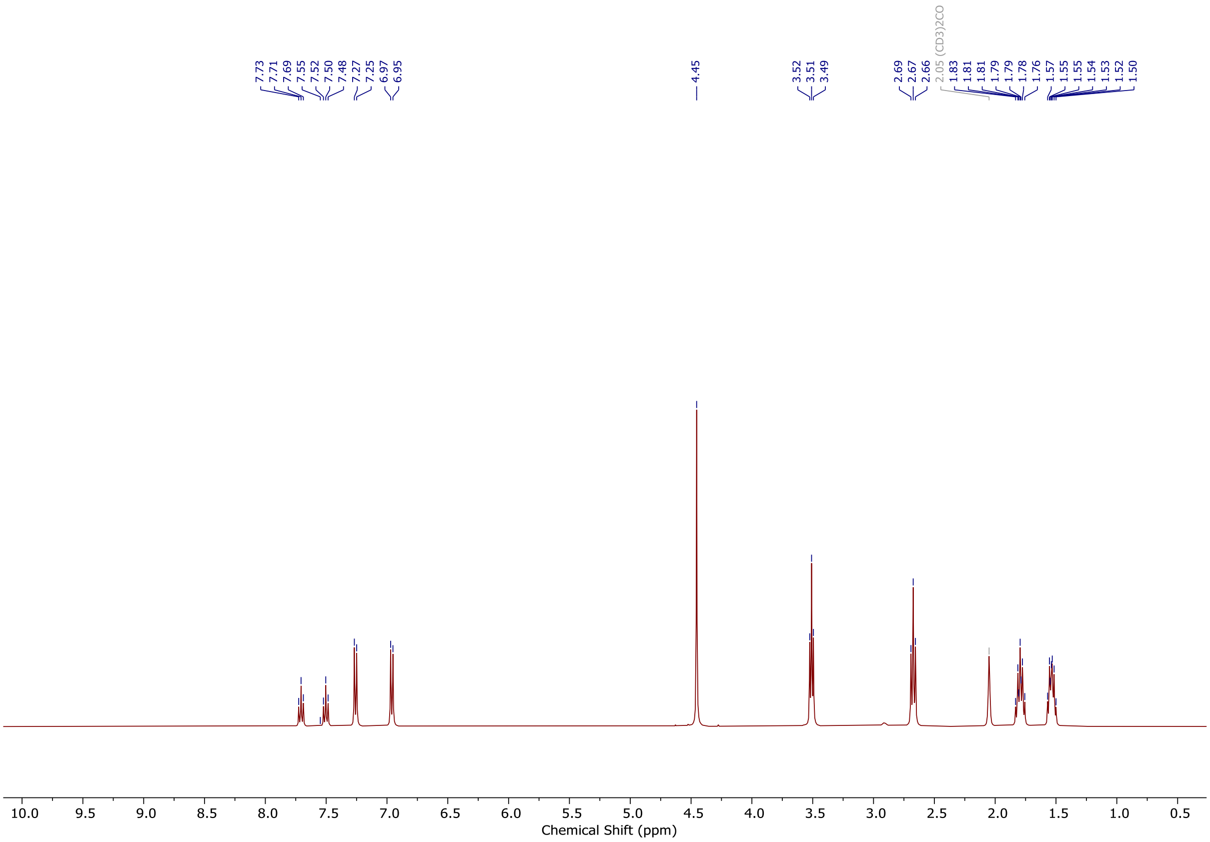

Figure S7. ^{1}H NMR spectrum of X (acetone-d_6, 400 MHz, 298 K).

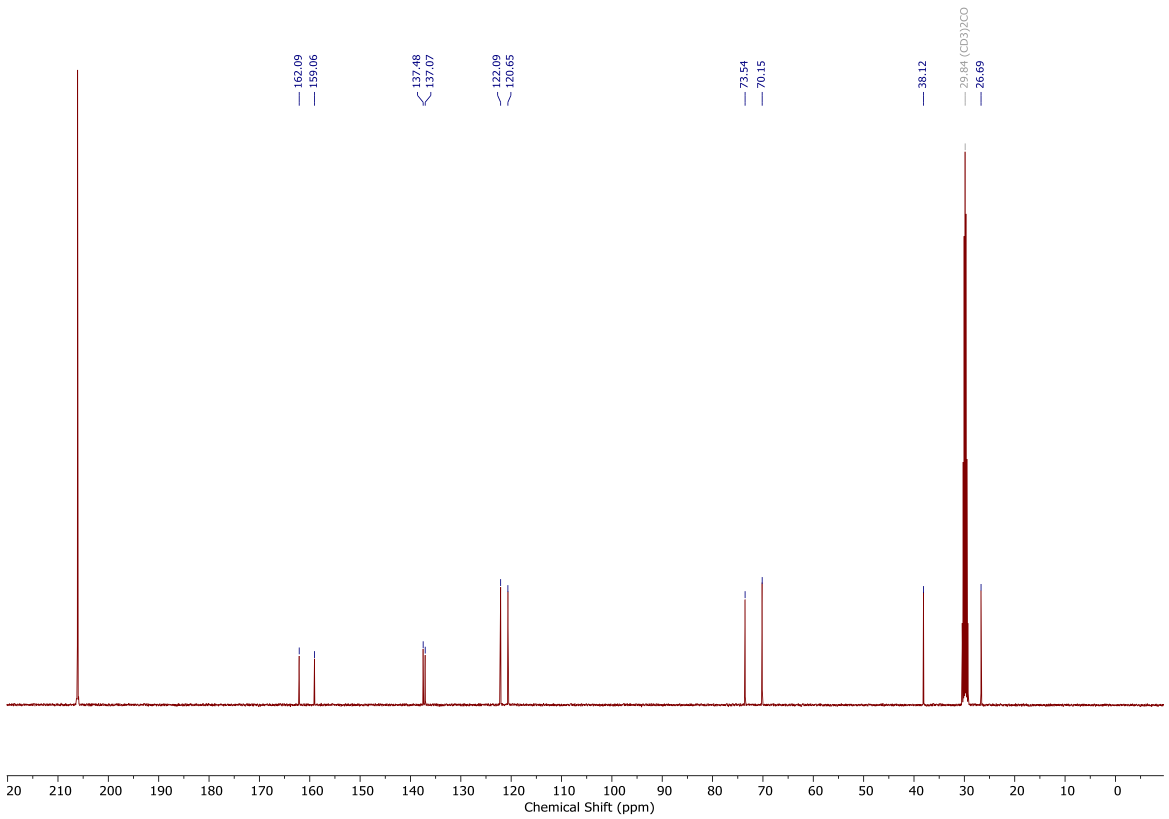

Figure S8. ^{13}C NMR spectrum of X (acetone-d_6, 101 MHz, 298 K).

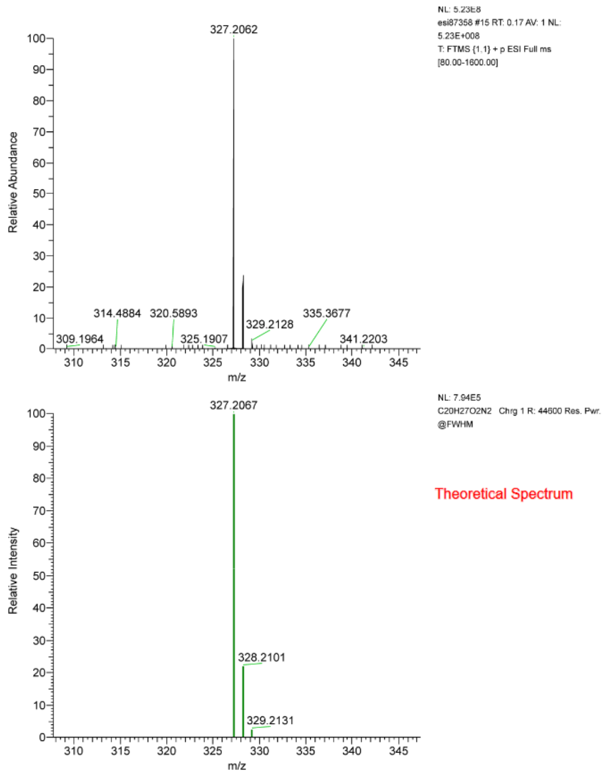

Figure S9. HRESI spectrum of X.

Characterisation of Br(I) Ag(I) and Au(I) complexes

As highlighted by Erdelyi *et al.* the characterisation of halenium complexes in general is challenging and ^{1}H and ^{13}C NMR spectroscopy is insufficient for characterisation, notwithstanding this for the purposes of completeness below is a summarised collection of the ^{1}H and ^{13}C NMR spectra for [**MC2**·$BrPF_6$], [**MC2**·$BrBF_4$], [**MC2**·$BrNO_3$], [**MC2**·$AgPF_6$], [**MC2**·$AgBF_4$], [**MC2**·$AgNO_3$], [**MC2**·$AuPF_6$], [**MC2**·$AuBF_4$], [**MC2**·$AuNO_3$]:

[**MC2**·$BrPF_6$]:

^{1}H NMR (400 MHz, CD_2Cl_2) δ 7.99 (s, 2H), 7.53 (s, 2H), 7.45 (d, *J* = 7.8 Hz, 2H), 4.73 (s, 4H), 3.82 (s, 4H), 3.27 – 2.99 (m, 4H), 2.04 (s, 4H), 1.82 (s, 4H).

^{13}C NMR-{^{1}H} (101 MHz, CD_2Cl_2) δ 163.09, 159.86, 137.32, 137.17, 121.93, 120.72, 73.81, 70.19, 38.17, 26.34.

[**MC2**·$BrBF_4$]:

^{1}H NMR (400 MHz, CD_2Cl_2) δ 7.97 (s, 2H), 7.49 (s, 2H), 7.44 (d, *J* = 7.7 Hz, 2H), 4.69 (s, 4H), 4.01 (s, 4H), 3.29 – 2.97 (m, 4H), 2.05 (s, 4H), 1.84 (s, 4H).

^{13}C NMR-{^{1}H} (101 MHz, CD_2Cl_2) δ 163.19, 159.76, 137.02, 137.17, 122.01, 120.80, 73.93, 70.21, 38.14, 26.29.

[**MC2**·$BrNO_3$]:

^{1}H NMR (400 MHz, CD_2Cl_2) δ 8.01 (s, 2H), 7.56 (s, 2H), 7.41 (d, *J* = 7.8 Hz, 2H), 4.71 (s, 4H), 4.04 (s, 4H), 3.31 – 2.99 (m, 4H), 2.10 (s, 4H), 1.85 (s, 4H).

^{13}C NMR-{^{1}H} (101 MHz, CD_2Cl_2) δ 163.20, 159.80, 137.22, 137.19, 121.98, 120.0, 74.24, 70.26, 38.15, 26.31.

[**MC2**·$AgPF_6$]:

^{1}H NMR (400 MHz, CD_2Cl_2) δ 7.93 (t, *J* = 7.7 Hz, 1H), 7.79 (t, *J* = 7.7 Hz, 1H), 7.43 (d, *J* = 7.7 Hz, 2H), 7.28 (d, *J* = 7.7 Hz, 2H), 4.66 (s, 4H), 3.84 (t, *J* = 5.7 Hz, 4H), 3.05 – 2.88 (m, 4H), 2.09 – 1.91 (m, 4H), 1.82 (p, *J* = 5.9 Hz, 4H).

^{13}C NMR-{^{1}H} (101 MHz, CD_2Cl_2) δ 163.19, 159.90, 137.12, 137.47, 122.01, 120.52, 73.71, 70.21, 38.19, 26.29.

[**MC2**·$AgBF_4$]:

^{1}H NMR (400 MHz, CD_2Cl_2) δ 7.94 (t, *J* = 7.7 Hz, 1H), 7.79 (t, *J* = 7.7 Hz, 1H), 7.44 (d, *J* = 7.7 Hz, 2H), 7.30 (d, *J* = 7.7 Hz, 2H), 4.62 (s, 4H), 3.90 (t, *J* = 5.8 Hz, 4H), 3.10 – 2.91 (m, 4H), 2.12 – 1.95 (m, 4H), 1.87 (p, *J* = 5.5 Hz, 4H).

^{13}C NMR-{^{1}H} (101 MHz, CD_2Cl_2) δ 163.21, 159.90, 137.21, 137.50, 122.13, 121.02, 74.09, 70.19, 38.21, 26.31.

[**MC2**·$AgNO_3$]:

^{1}H NMR (400 MHz, CD_2Cl_2) δ 7.99 (m, 1H), 7.81 (m, 1H), 7.43 (d, *J* = 7.7 Hz, 2H), 7.30 (d, *J* = 7.7 Hz, 2H), 4.63 (s, 4H), 3.91 (t, *J* = 5.8 Hz, 4H), 3.12 – 2.92 (m, 4H), 2.11 – 1.99 (m, 4H), 1.89 (p, *J* = 5.5 Hz, 4H).

^{13}C NMR-{^{1}H} (101 MHz, CD_2Cl_2) δ 163.22, 159.91, 137.24, 137.48, 122.17, 121.07, 74.11, 70.17, 38.24, 26.34.

[**MC2**·$AuPF_6$]:

^{1}H NMR (400 MHz, CD_2Cl_2) δ 7.94 (t, *J* = 7.8 Hz, 1H), 7.81 (t, *J* = 7.8 Hz, 1H), 7.44 (d, *J* = 7.8 Hz, 2H), 7.30 (d, *J* = 7.8 Hz, 2H), 4.63 (s, 4H), 3.82 (t, *J* = 5.7 Hz, 4H), 3.09 – 2.86 (m, 4H), 2.11 – 1.92 (m, 4H), 1.83 (p, *J* = 5.9 Hz, 4H).

^{13}C NMR-{^{1}H} (101 MHz, CD_2Cl_2) δ 163.21, 159.91, 137.17, 137.44, 122.08, 120.54, 73.72, 70.27, 38.21, 26.31.

[**MC2**·$AuBF_4$]:

^{1}H NMR (400 MHz, CD_2Cl_2) δ 7.93 (t, *J* = 7.8 Hz, 1H), 7.80 (t, *J* = 7.8 Hz, 1H), 7.47 (d, *J* = 7.8 Hz, 2H), 7.29 (d, *J* = 7.8 Hz, 2H), 4.67 (s, 4H), 3.87 (t, *J* = 5.7 Hz, 4H), 3.10 – 2.84 (m, 4H), 2.12 – 1.94 (m, 4H), 1.81 (p, *J* = 5.9 Hz, 4H).

^{13}C NMR-{^{1}H} (101 MHz, CD_2Cl_2) δ 163.20, 159.94, 137.19, 137.41, 122.10, 120.41, 73.42, 70.29, 38.19, 26.29.

[**MC2**·$AuNO_3$]:

^{1}H NMR (400 MHz, CD_2Cl_2) δ 7.91 (m, 1H), 7.79 (m, 1H), 7.49 (d, *J* = 7.8 Hz, 2H), 7.31 (d, *J* = 7.8 Hz, 2H), 4.69 (s, 4H), 3.81 (t, *J* = 5.7 Hz, 4H), 3.11 – 2.80 (m, 4H), 2.13 – 1.94 (m, 4H), 1.87 (p, *J* = 5.9 Hz, 4H).

^{13}C NMR-{^{1}H} (101 MHz, CD_2Cl_2) δ 162.90, 160.08, 137.24, 137.21, 122.19, 120.47, 73.44, 70.31, 38.21, 26.31.

3. Crystal Structure Determination

Single crystals of [**MC1**·$AgPF_6$], [**MC1**·$AuPF_6$], [**MC2**·$AgPF_6$], [**MC2**·$AuPF_6$], [**MC2**·$AgBF_4$], [**MC2**·$AgNO_3$], [**MC2**·$AuBF_4$], [**MC2**·$AuNO_3$], [**MC2**·$BrPF_6$], [**MC2**·$BrBF_4$] and [**MC2**·$BrNO_3$] suitable for X-ray analysis were each coated with Paratone-N oil, suspended on a 200 μm MiTeGen loop, and placed in a cold gaseous nitrogen stream on an Oxford Diffraction Supernova X-ray diffractometer performing φ- and ω-scans at 150(2) K. Diffraction16 intensities were measured using graphite monochromated Cu K_{α} radiation (1.54184 Å). Data collection, indexing, initial cell refinements, frame integration, final cell refinements and absorption corrections were accomplished using the program CrysAlisPro.† Scattering factors and anomalous dispersion corrections were taken from the International Tables for X-ray Crystallography. All structures were solved by direct methods and refined against F2. All hydrogen atoms were included into the model at geometrically calculated positions and refined using a riding model. Details of the data quality and a summary of the residual values for the refinements are listed in Table S1. Figures of the crystal structures have been created using the open-source PyMOL Molecular Graphics System.

Deposition Numbers: 2371354 (for [**MC1**·$AgPF_6$]), 2371337 (for [**MC1**·$AuPF_6$]), 2371333 (for [**MC2**·$AgPF_6$]), 2371340 (for [**MC2**·$AuPF_6$]), 2371338 (for [**MC2**·$AgBF_4$]), 2371339 (for [**MC2**·$AgNO_3$]), 2371338 (for [**MC2**·$AuBF_4$]), 2371335 (for [**MC2**·$AuNO_3$]), 2371336 (for [**MC2**·$BrPF_6$]), 2371341 (for [**MC2**·$BrBF_4$]) and 2371355 (for [**MC2**·$BrNO_3$]) contains the supplementary crystallographic data for this paper. This data is provided free of charge by the joint Cambridge Crystallographic Data Centre and Fachinformationszentrum Karlsruhe Access Structures service www.ccdc.cam.ac.uk/structures.

† CrysAlisPRO, Oxford Diffraction /Agilent Technologies UK Ltd, Yarnton, England.

Table S1. Selected crystallographic data for **MC1** complexes co-crystallised with PF_6^-.

**Compound**	**MC1·AgPF$_6$**	**MC1·AuPF$_6$**
**Formula**	C_{22} H_{30} Ag N_2, F_6 P, C H_2 Cl_2	C_{22} H_{30} Au N_2, F_6 P, C H_2 Cl_2
**Formula Weight**	660.25	749.35
**a (Å)**	9.0869(1)	12.0937(1)
**b (Å)**	19.6623(2)	10.9701(1)
**c (Å)**	15.4434(1)	20.5471(2)
***α* (º)**	90	90
***β* (º)**	105.8107(9)	105.1837(8)
***γ* (º)**	90	90
**Unit cell volume (Å^3)**	2654.87(4)	2630.80(4)
**Crystal system**	Monoclinic	Monoclinic
**Space group**	P 21/c	P 21/n
**Z**	4	4
**Temperature (K)**	150 K	150 K
**Radiation Type**	Copper	Copper
**λ (Å)**	1.54184	1.54184
**Reflections (all)**	14931	14557
**Reflections (unique)**	5506	5443
**R_{int}**	0.022	0.022
**R[I > 2σ(I)]**	0.0299	0.0242
**wR(F^2) (all)**	0.0745	0.0630
**S**	1.007	0.998

Table S2. Selected crystallographic data for **MC2** complexes co-crystallised with PF_6^-.

**Compound**	**MC2·BrPF$_6$**	**MC2·AgPF$_6$**	**MC2·AuPF$_6$**
**Formula**	C_{20} H_{26} Br N_2 O_2, F_6 P	C_{20} H_{26} Ag N_2 O_2, F_6 P	C_{20} H_{26} Au N_2 O_2, F_6 P
**Formula Weight**	551.30	579.27	668.37
**a (Å)**	11.5204(2)	9.4670(1)	11.4974(2)
**b (Å)**	9.4275(2)	11.4980(1)	9.3872(2)
**c (Å)**	20.2367(4)	19.8471(2)	20.2176(5)
α **(°)**	90	90	90
β **(°)**	97.6240(18)	90	98.060(2)
γ **(°)**	90	90	90
**Unit cell volume (Å^3)**	2178.45(7)	2160.39(4)	2160.50(8)
**Crystal system**	Monoclinic	Orthorhombic	Monoclinic
**Space group**	P 21/n	P n m a	P 21/n
**Z**	4	4	4
**Temperature (K)**	150 K	150 K	150 K
**Radiation Type**	Copper	Copper	Copper
**λ (Å)**	1.54184	1.54184	1.54184
**Reflections (all)**	11115	47336	10950
**Reflections (unique)**	4498	2386	4477
**R_{int}**	0.029	0.045	0.034
**R[I > 2σ(*I*)]**	0.0437	0.0239	0.0350
**wR(F^2) (all)**	0.1160	0.0643	0.0940
**S**	1.023	1.003	1.013

Table S3. Selected crystallographic data for **MC2** complexes co-crystallised with BF_4^-.

Compound	MC2·BrBF$_4$	MC2·AgBF$_4$	MC2·AuBF$_4$
**Formula**	C_{20} H_{26} Br N_2 O_2, B F_4	C_{20} H_{26} Ag N_2 O_2, B F_4	C_{20} H_{26} Au N_2 O_2, B F_4
**Formula Weight**	493.15	521.11	610.21
**a (Å)**	11.2164(3)	11.0544(1)	11.2013(2)
**b (Å)**	9.2532(3)	9.3938(1)	9.2294(1)
**c (Å)**	20.0349(5)	19.6489(2)	19.8827(2)
α **(°)**	90	90	90
β **(°)**	96.530(2)	96.9146(8)	97.6829(13)
γ **(°)**	90	90	90
**Unit cell volume (Å^3)**	2065.88(10)	2025.56(4)	2037.05(5)
**Crystal system**	Monoclinic	Monoclinic	Monoclinic
**Space group**	P 21/n	P 21/n	P 21/n
**Z**	4	4	4
**Temperature (K)**	150 K	150 K	150 K
**Radiation Type**	Copper	Copper	Copper
**λ (Å)**	1.54184	1.54184	1.54184
**Reflections (all)**	11192	14225	10425
**Reflections (unique)**	4265	4189	4204
**R_{int}**	0.065	0.025	0.023
**R[I > 2σ(I)]**	0.0778	0.0237	0.0267
**wR(F^2) (all)**	0.1606	0.0604	0.0710
**S**	1.074	1.028	0.999

Table S4. Selected crystallographic data for **MC2** complexes co-crystallised with NO_3^-.

Compound	MC2·BrNO$_3$	MC2·AgNO$_3$	MC2·AuNO$_3$
Formula	C_{20} H_{26} Br N_2 O_2, N O_3	C_{20} H_{26} Ag N_2 O_2, N O_3	C_{20} H_{26} Au N_2 O_2, N O_3
Formula Weight	468.35	496.31	585.41
a (Å)	10.9813(2)	12.4752(1)	11.0010(2)
b (Å)	9.0131(2)	17.6251(1)	9.0134(2)
c (Å)	20.1527(4)	27.6185(1)	20.0199(4)
α (º)	90	90	90
β (º)	94.9674(17)	90	96.5686(17)
γ (º)	90	90	90
Unit cell volume (Å^3)	1987.13(7)	6072.66(6)	1972.07(7)
Crystal system	Monoclinic	Orthorhombic	Monoclinic
Space group	P 21/n	P 21 21 21	P 21/n
Z	4	12	4
Temperature (K)	150 K	150 K	150 K
Radiation Type	Copper	Copper	Copper
λ (Å)	1.54184	1.54180	1.54180
Reflections (all)	10588	167574	21338
Reflections (unique)	4104	12689	4087
R_{int}	0.030	0.036	0.054
R[I > 2σ(*I*)]	0.0389	0.0185	0.0426
wR(F^2) (all)	0.1108	0.0488	0.1224
S	1.014	1.010	1.007

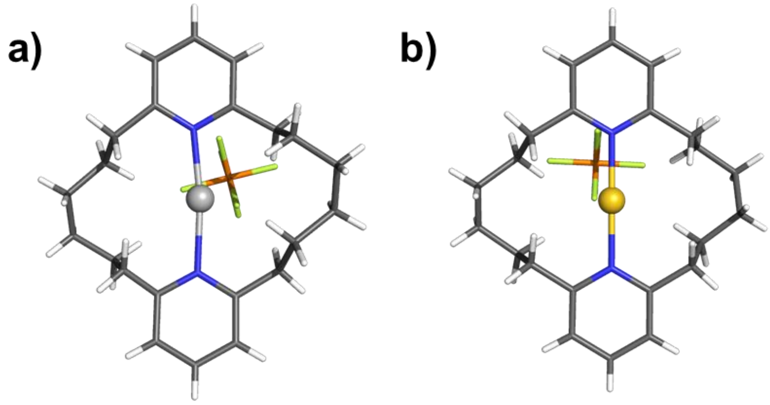

Figure S10. X-ray Crystal Structures of a) [**MC1**·$AgPF_6$] and b) [**MC1**·$AuPF_6$].

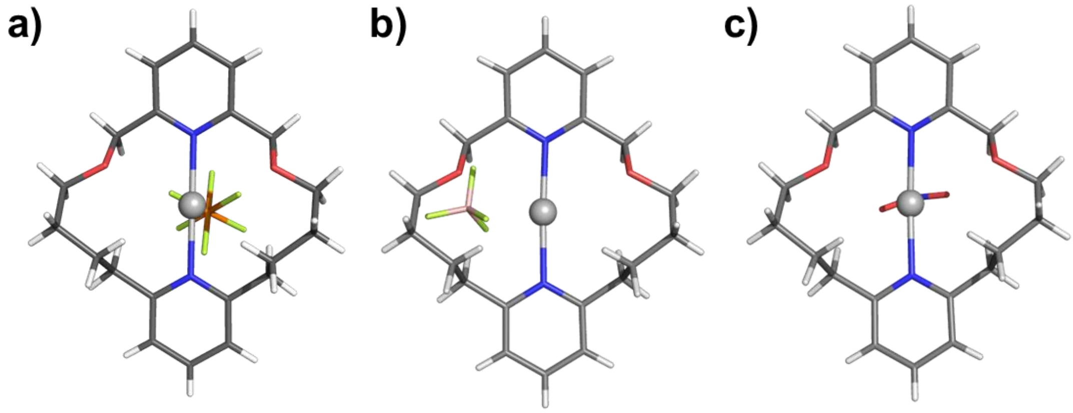

Figure S11. X-ray Crystal Structures of a) [**MC2**·$AgPF_6$] b) [**MC2**·$AgBF_4$] c) [**MC2**·$AgNO_3$].

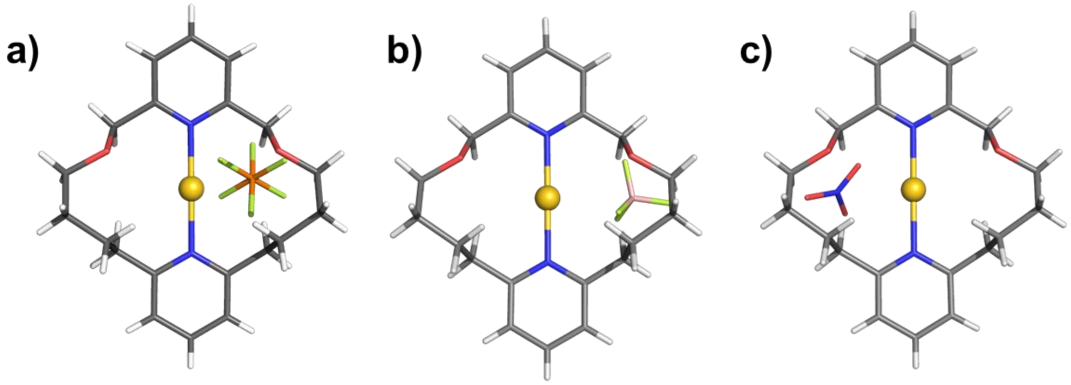

Figure S12. X-ray Crystal Structures of a) [**MC2**·$AuPF_6$] b) [**MC2**·$AuBF_4$] c) [**MC2**·$AuNO_3$].

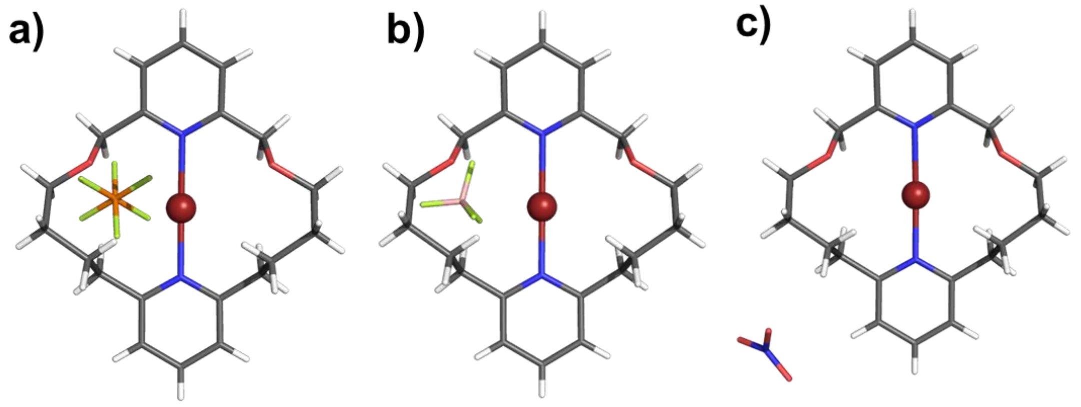

Figure S13. X-ray Crystal Structures of a) [**MC2**·$BrPF_6$] b) [**MC2**·$BrBF_4$] c) [**MC2**·$BrNO_3$].

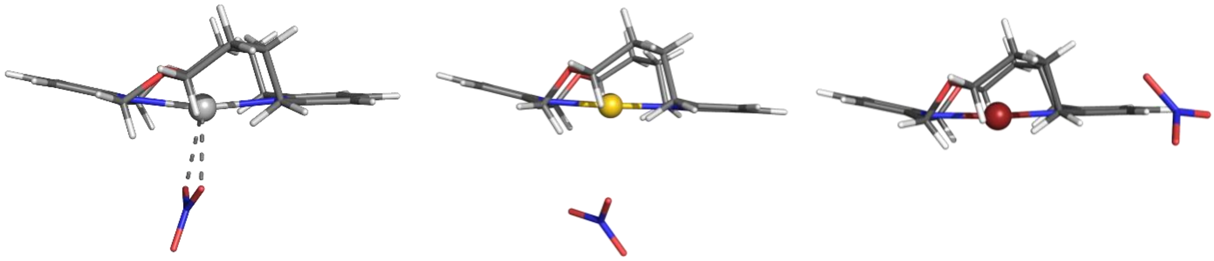

Figure S14. X-ray single crystal structures of a) [**MC2**·$AgNO_3$], b) [**MC2**·$AuNO_3$] and c) [**MC2**·$BrNO_3$].
